# When Combined with Colistin, an Otherwise Ineffective Rifampicin–Linezolid Combination Becomes Active in *Escherichia coli*, *Pseudomonas aeruginosa*, and *Acinetobacter baumannii*

**DOI:** 10.3390/microorganisms8010086

**Published:** 2020-01-08

**Authors:** Eva Armengol, Teresa Asunción, Miguel Viñas, Josep Maria Sierra

**Affiliations:** Laboratory of Molecular Microbiology and Antimicrobials, Department of Pathology and Experimental Therapeutics, School of Medicine. Institut d’Investigacions biomèdiques de Bellvitge (IDIBELL), University of Barcelona, 08907 L’Hospitalet, Barcelona, Spain; eva.armengol.rivero@gmail.com (E.A.); teresa.asuncion.escorsa@gmail.com (T.A.); mvinyas@ub.edu (M.V.)

**Keywords:** antibiotic combinations, synergism, multidrug-resistant (MDR) bacteria

## Abstract

The synergistic action of colistin, with two antibiotics active in Gram-positive bacteria but unable to kill gram negatives (linezolid and rifampicin), was investigated, since triple combinations are emerging as a tool to overtake multidrug resistance. Checkerboard determinations demonstrated that, when combined with colistin, the combination of linezolid and rifampicin turns active in multidrug-resistant *Escherichia coli*, *Pseudomonas aeruginosa*, and *Acinetobacter baumannii*. Thus, the presence of sublethal concentrations of colistin resulted in a strongly synergistic interaction between these two drugs. Moreover, the minimum inhibitory concentrations of linezolid–rifampicin combinations in the presence of colistin were lower than the maximal concentrations of these antimicrobials ain blood. These findings suggest the use of this triple combination as an effective treatment of multidrug-resistant (MDR) bacterial infections.

## 1. Introduction

The mechanisms of action of cationic antimicrobial peptides (CAMPs) are not fully understood, but the ability of these drugs to interact with the bacterial membrane and thus alter its permeability is well established. CAMPs are thought to induce the formation of pore-forming channels, as described by barrel-stave, toroidal pore, carpet, and aggregate models [1], thus facilitating the entry of other antimicrobials into the bacterial cell interior. Synergies between different CAMPs and between CAMPs and classical antibiotics may therefore offer methods for the re-sensitization of bacteria that have become resistant to several antibiotics [2,3,4].

Colistin is a CAMP whose clinical use was abandoned due to its nephro and neurotoxicity. However, with the emergence of multidrug-resistant (MDR) bacteria and the need for effective antimicrobials, the therapeutic potential of colistin is being re-evaluated. Colistin’s main mechanism of action is the disruption of bacterial membranes, leading to severe alterations involving their permeability [5]. Since drug resistance is often totally or partially due to a restricted entry or enhanced efflux, low concentrations of colistin have been used to sensitize bacteria to different antimicrobials [3]. Specifically, in vitro synergies [6] between low-dose colistin and other antimicrobials have been demonstrated in *Escherichia coli* [7], *Pseudomonas aeruginosa* [8], and *Acinetobacter baumannii* [9].

Despite these positive results, the increase in emerging MDR bacteria has shown that the combined use of two antimicrobial agents may not be enough to effectively stop these infections. Instead, the use of triple antimicrobial combinations has been suggested, based on encouraging in vitro data [10,11]. For example, triple antimicrobial therapies consisting of gentamicin–carbenicillin–rifampin and dicloxacillin–fusidic acid–rifampicin have been proposed for the treatment of *Stenotrophomonas maltophilia* and *Staphylococcus aureus* infections, respectively [12,13,14,15]. Colistin-based combinations were assessed by Huang et al. [11], who concluded that they provide good clinical treatment options.

Analogous to the combined use of two antimicrobials, the goal of triple antimicrobial therapy is to increase the activity of the antimicrobial agents and circumvent the development of antimicrobial resistance. As noted above, colistin is able to enhance the antimicrobial action of other antibiotics, even when used in very low concentrations [2]. It weakens the permeability barrier of the outer membrane, which otherwise limits the action of many antimicrobials. In addition, low-dose colistin was shown to inhibit bacterial efflux pumps [3]. Thus, in this study, we examined the ability of colistin to activate linezolid and rifampicin in *E. coli*, *P. aeruginosa*, and *A. baumannii*, since almost every strain is intrinsically resistant to both agents.

Linezolid is considered active only in gram positives. The reason is that the molecule is expelled by an efflux pump in gram negatives. It has been shown that colistin increases permeability, not only by altering the bilayer structure but also by acting on membrane fluidity, having an effect on the reduction of efflux pumps activities (3). A major challenge of microbiology is to present new ways to treat infections caused by MDR Gram-negative bacteria. In this work, we explore if a combination with low concentrations of colistin results in sensitization against linezolid. Furthermore, as triple combinations are being regarded as a useful strategy, not only to enhance antimicrobial action but to slow down the migration from susceptibility to resistance, the effect of triple combinations including colistin and linezolid are investigated.

## 2. Materials and Methods 

### 2.1. Bacterial Strains, Culture Conditions, and Chemicals

Twenty-three MDR clinical isolates, of which eight were *E. coli*, seven were *P. aeruginosa*, and seven were *A. baumannii*, which differed in their clinical origins and were epidemiologically unrelated, were used in this study. The control strains used were *E. coli* ATCC 25922, *P. aeruginosa* ATCC 27853 and PAO1, and *A. baumannii* ATCC 17978. The strains were cultured 24 h at 37 °C in tryptone soy agar, tryptone soy broth, and Mueller–Hinton broth, all of which were purchased from Sharlau (Sentmenat, Barcelona, Spain).

In this study, three different antibiotic substances were used. Colistin was kindly supplied by Zhejiang Shenghua Biok Biology Co., Ltd., (Shangai, China). Linezolid and rifampin were purchased from Sigma–Aldrich Chemicals (Madrid, Spain). 

### 2.2. Susceptibility Test

The minimum inhibitory concentrations (MICs) of colistin, rifampicin, and linezolid were determined using the microdilution method, according to EUCAST recommendations [16]. As rifampicin and linezolid breakpoints for Gram-negative bacteria are not defined by EUCAST, susceptibility could not be assessed. 

### 2.3. Checkerboard Assay

Firstly, checkerboards of colistin–rifampin, colistin–linezolid, and rifampin–linezolid were performed for each strain using doubling dilutions. Fractional inhibitory concentration index (FICI) values were calculated as

$$\begin{array}{ll} FICI= & FIC\;A\;\left(MIC\;antibiotic\;A\;in\;combination\right)/\left(MIC\;antibiotic\;A\right)\\ & +\;FIC\;B\;\left(MIC\;antibiotic\;B\;in\;combination\right)/\left(MIC\;antibiotic\;B\right) \end{array}$$

Synergy was defined as a FICI <0.5, no interaction as a FICI between 0.5 and 4, and antagonism as a FICI of 4 or higher.

Furthermore, the checkerboard of rifampin–linezolid was performed by adding colistin at ½ MIC of each strain. In the horizontal wells, rifampin (0.06–64 mg/L) was set, and linezolid (0.5–32 mg/L) was set in the vertical wells. The FICI values obtained for the linezolid–rifampin combinations in the presence and absence of ½ MIC colistin were compared. 

All experiments were done in triplicate.

## 3. Results

Susceptibility Test and Checkerboard Assay

In order to analyze the combination rifampin–linezolid with subinhibitory concentrations of colistin, susceptibility tests and checkerboard assays were performed in *E. coli*, *P. aeruginosa*, and *A. baumannii* strains. In Table 1, the results of the rifampin, linezolid, and colistin combinations in *E. coli* are shown. All the strains were susceptible to colistin, with concentrations ranging from 0.25 mg/L to 1 mg/L. The MICs of rifampin and linezolid were 4–8 mg/L and ~256 mg/L, respectively. In all the strains, FICIs around 0.625 for the combination of linezolid–colistin were determined, demonstrating that there was not synergy between the antibiotics, even though the linezolid MIC decreased. In five strains, synergy was seen for rifampin–colistin combinations, with FICIs from 0.151 to 0.312. The combination of rifampin–linezolid did not present any synergy with FICIs above 1. When ½ MIC of colistin was added to the combination, a synergistic effect was apparent in all the strains, with FICIs ranging from 0.012 to 0.265 (Table 2). In summary, subinhibitory concentrations of colistin turn resistant *E. coli* strains susceptible. 

The results of combinations on *P. aeruginosa* are presented in Table 3 and Table 4. The MIC of colistin ranged from 1 mg/L to 0.5 mg/L in *P. aeruginosa* strains, presenting a susceptible profile. Rifampin MICs were more variable, showing concentrations from 64 mg/L to 8 mg/L. In contrast, linezolid MICs were mostly 256 mg/L. The checkerboard assay of combinations rifampin–colistin, linezolid–colistin, and rifampin–linezolid did not show any synergy with FICIs between 0.625 and 2.125. In contrast, when subinhibitory concentrations of colistin were added to a rifampin–linezolid combination, the FICIs decreased dramatically, ranging from 0.010 to 0.266. 

All the strains of *A. baumannii* resulted in susceptibility to colistin, with MICs of 1 mg/L and 0.5 mg/L (Table 5). For rifampicin, the strains presented different MIC values. Four strains show relatively low values (2 mg/L and 4 mg/L), while the other four had high values (32 mg/L and 64 mg/L). The combination of colistin–rifampin resulted in synergy for two strains, with FICIs ranging from 0.375 to 0.265, while the other six did not. No synergism was detected for combinations of colistin–linezolid and rifampin–linezolid, with FICIs from 0.531 to 2.125 (Table 6). Nevertheless, as with the other two bacterial species tested, when subinhibitory colistin concentrations were added to the rifampin–linezolid combination, the FICIs decreased greatly, as shown in Table 6 i.e. combinations were strongly synergistic. 

## 4. Discussion

Infections caused by MDR bacteria are responsible for ~70,000 deaths/year, and that number is expected to reach 10 million deaths/year by 2050 [17,18]. Several national and international programs have been undertaken to address this crisis, including the European Union’s Innovative Medicine Initiative (IMI), the Joint Programming Initiative on Antimicrobial Resistance (JPIAMR), and the Plan Nacional de Resistencia a los Antibióticos (PRAN) in Spain.

Among the proposed strategies is to combine two or more already known antimicrobial agents to increase their activities compared to the respective single antimicrobials. An additional advantage of this approach is the avoidance or minimization of the emergence of resistant variants. Based on their mechanisms of action, CAMPs are good candidate drugs likely to increase the efficacy of these combinations [19].

In this study, we explored possible synergies between CAMPs and antimicrobials lacking activity in Gram-negative bacteria [20,21]; specifically, the triple antimicrobial combination of colistin–-rifampicin–linezolid. Greater activity of both colistin–rifampicin [9] and colistin–linezolid [20] than either rifampicin or linezolid alone has been reported. Based on previous reports of the ability of sublethal concentrations of colistin to permeabilize bacterial membranes, we tested colistin in combination with rifampicin and linezolid, two antimicrobials otherwise ineffective in treating *E. coli* infections. The idea was to use colistin as an enhancer, rather than an antibiotic itself, to activate the two other drugs that alone have no activity. 

Thus, the presence of sublethal concentrations of colistin resulted in a strongly synergistic interaction between these two drugs. Moreover, the MICs of linezolid–rifampicin combinations in the presence of colistin were lower than the maximal concentrations of these antimicrobials achievable in blood. These findings suggest the use of this triple combination as an effective treatment of MDR bacterial infections. 

Our results demonstrate that colistin, and presumably other CAMPs, can act as permeabilizers that potently increase the synergism of linezolid and rifampicin, and perhaps of other combinations of antimicrobials [22]. The linezolid resistance of Gram-negative bacteria is mainly due to the inability of the antimicrobial to reach effective intracytoplasmic concentrations. In *E. coli*, this is because linezolid is a good substrate of MDR efflux pumps, such as AcrAB-TolC [23]. However, in the presence of sublethal concentrations of colistin, the function of the bacterial efflux machinery is seriously compromised [3], such that linezolid is able to enter the cell in concentrations high enough to inhibit protein synthesis. 

The use of antimicrobial agents in triple combinations has been explored in several recent studies [10,24,25]. As in our study, most of them included colistin, and the results were similar to ours. The use of triple combinations containing colistin can therefore be recommended as a novel potential treatment for MDR infections caused by *E. coli*, *P. aeruginosa*, and *A baumannii* strains. 

Tsala et al. [14] described the bactericidal activity of the triple combination meropenem–tigecycline–colistin against carbapenem-producing *Klebsiella pneumoniae*. In a checkerboard analysis, these antimicrobials showed additive interactions. The efficacy of the triple combination was demonstrated in an in vitro dynamic PK/PD model, in which two different dose regimens of tigecycline were tested. The most pronounced effects were obtained using the higher dose.

Li et al. [10] reported the synergistic activity of rifampicin–azithromycin–colistin against *E. coli* producing Mcr-1. This triple antimicrobial combination was tested in vitro (time kill curves) and in an animal model (neutropenic mouse thigh model). The triple combination was more effective than treatment with any of the antimicrobials alone or with double antimicrobial treatments.

In conclusion, colistin-based triple antimicrobial combinations may offer a promising alternative in the treatment of infections by MDR bacteria. Our study specifically demonstrated the potential efficacy of a combination of colistin, linezolid, and rifampicin in Gram-negative bacterial MDR strains. As has been mentioned, cationic peptides may sensitize bacteria to a wide variety of antibiotics in which the bacteria have acquired some mechanism of resistance involving permeability. In our work, we succeeded in increasing susceptibility of three bacterial species intrinsically resistant to linezolid. This sensitization was particularly drastic when measuring synergisms between linezolid and rifampin. In other words, the criterion that the linezolid spectrum of activity is restricted to in all clinically important Gram-positive bacteria, such as *Enterococcus faecium* and *E. faecalis* (including vancomycin-resistant enterococci); *S. aureus* (including methicillin-resistant *S. aureus*, MRSA); and other streptococci, such as *Listeria* or *Corynebacterium*, should be extended. When CAMPS (like colistin) were combined with other antimicrobials (linezolid, in our case), they made otherwise fully and constitutively resistant Gram-negative bacteria susceptible. However, the in vivo efficacy of this approach must still be confirmed in further studies, including animal models. While the mechanism of action may be directly related to the ability of colistin to allow other antimicrobials to penetrate the bacterial envelope, the inhibition of efflux pumps as a consequence of the membrane damage induced by colistin cannot be ruled out. In fact, there is increasing evidence that colistin can enhance the activities of a large number of antimicrobials [26,27] or enlarge their spectrum of action [28].

## Figures and Tables

**Table 1:** Minimum inhibitory concentrations (MICs) according to EUCAST [16] and values for the combination of rifampin–linezolid with subinhibitory concentrations of colistin.

MIC							
STRAIN	COL	RIF	LNZ	RIF/COL	LNZ/COL	RIF/LNZ	RIF/LNZ + ½MIC COL
MDR 211453	0.5	8	256	16/0.25	32/0.25	16/32	0.5/4
MDR 39255	0.5	8	256	16/0.25	32/0.25	16/32	0.03/2
MDR 208691	0.25	8	256	16/0.125	32/0.125	8/32	2/4
MDR 205119	0.5	8	128	0.25/0.125	16/0.25	8/32	2/1
MDR 246415	1/0.5	8	256	0.25/0.06	32/0.5	8/32	0.5/2
MDR 239910	1/0.5	8	256	4/0.125	16/0.5	16/32	0.5/1
MDR 238192	0.5/0.25	8	256	0.5/0.125	16/0.25	8/32	0.03/1
MDR 215482	1	8	>256	0.25/0.25	64/0.5	8/32	0.5/4
ATCC 29255	0.5	4	128	16/0.25	16/0.25	8/32	0.5/1

COL = colistin, RIF = rifampin, and LNZ = linezolid. MICs are in mg/L.

**Table 2:** Checkerboards of the different antibiotics used with their fractional inhibitory concentration indexes (FICIs) in multidrug-resistant (MDR) *E. coli* strains.

FICI				
STRAIN	RIF + COL	LNZ + COL	RIF + LNZ	RIF + LNZ + ½MIC COL
MDR 211453	2.5	0.625	2.125	0.078
MDR 39255	2.5	0.625	2.125	0.012
MDR 208691	2.5	0.625	1.125	0.265
MDR 205119	0.281	0.625	1.125	0.258
MDR 246415	0.151	0.625	1.125	0.070
MDR 239910	0.75	0.5625	2.125	0.066
MDR 238192	0.312	0.5625	1.125	0.008
MDR 215482	0.281	0.75	1.125	0.078
ATCC 29255	2.5	0.625	2.125	0.133

**Table 3:** MICs according to EUCAST [16] in MDR *P. aeruginosa* strains.

MIC							
STRAIN	COL	RIF	LNZ	RIF/COL	LNZ/COL	RIF/LNZ	RIF/LNZ + ½MIC COL
PA 362 VH	1	32	>256	16/0.5	64/0.5	32/32	4/2
PA 350 VH	0.5	8	256	1/0.25	32/0.25	8/32	0.125/1
PA 056 SJD	1	32	>256	8/0.5	32/0.5	64/32	0.063/2
PA 846 VH	0.5	8	128	1/0.25	16/0.25	16/32	2/2
PA 01	1	32	>256	4/0.5	32/0.5	32/32	4/4
PA 023 VH	1	64	>256	32/0.5	64/0.5	64/32	4/4
PA 666 SJD	1	32	>256	4/0.5	32/0.5	32/32	0.5/8
PA 086 SJD	1	16	>256	8/0.5	64/0.5	32/32	0.5/2
PA ATCC 27853	0.5	32	>256	32/0.25	64/.25	64/32	0.25/8

COL: Colistin; RIF: Rifampin; LNZ: Linezolid. MICs are in mg/L.

**Table 4:** Checkerboards of the different antibiotics used in this study, with their FICIs in MDR *P. aeruginosa* strains.

FICI				
STRAIN	RIF + COL	LNZ + COL	RIF + LNZ	RIF + LNZ + ½MIC COL
PA 362 VH	1	0.75	1.125	0.133
PA 350 VH	2.125	0.625	1.125	0.020
PA 056 SJD	0.75	0.625	2.125	0.010
PA 846 VH	0.625	0.625	2.25	0.266
PA 01	0.625	0.625	1.125	0.141
PA 023 VH	1	0.75	1.125	0.078
PA 666 SJD	0.625	0.625	1.125	0.047
PA 086 SJD	1	0.75	2.125	0.039
PA ATCC 27853	1.5	0.75	2.125	0.039

**Table 5:** MICs according to EUCAST [16] in MDR *A. baumannii* strains.

MIC							
STRAIN	COL	RIF	LNZ	RIF/COL	LNZ/COL	RIF/LNZ	RIF/LNZ + ½MIC COL
ABAU 137	1/0.5	64	>256	0.125/0.25	4/0.5	64/32	0.125/1
ABAU 80	1/0.5	2	>256	0.25/0.125	8/0.5	2/32	0.015/0.5
ABAU 226	1/0.5	2	>256	0.031/0.25	16/0.5	2/32	0.03/0.5
ABAU 8	1/0.5	32	>256	0.5/0.125	16/0.5	32/32	0.03/1
ABAU RUH-875	1/0.5	2	>256	0.5/0.125	16/0.5	2/32	0.0623/0.5
ABAU178	1/0.5	>64	>256	0.5/0.25	8/0.5	>64/32	0.5/4
ABAU 34	1/0.5	64	>256	0.125/0.25	16/0.5	64/32	0.125/2
ATCC 17978	0.5	4	>256	0.5/0.25	16/0.5	8/32	0.125/0.5

COL: Colistin; RIF: Rifampin; LNZ: Linezolid. MICs are in mg/L.

**Table 6:** Checkerboards of the different antibiotics used with their FICIs in MDR *A. baumannii* strains.

FICI				
STRAIN	RIF + COL	LNZ + COL	RIF + LNZ	RIF + LNZ + ½MIC COL
ABAU 137	0.502	0.516	1.125	0.006
ABAU 80	0.375	0.531	1.125	0.009
ABAU 226	0.5155	0.563	1.125	0.017
ABAU 8	0.265	0.563	1.125	0.005
ABAU RUH-875	0.5	0.563	1.125	0.033
ABAU 178	0.508	0.531	1.125	0.023
ABAU 34	0.502	0.563	1.125	0.010
ATCC 17978	0.625	0.563	2.125	0.033

COL: Colistin; RIF: Rifampin; LNZ: Linezolid. MICs are in mg/L.
